# Two new species of *Periconia* and a new record of *Paramonodictys* from *Miscanthus sinensis* in Guizhou Province, China

**DOI:** 10.3897/mycokeys.127.180874

**Published:** 2026-01-26

**Authors:** Li-Juan Zhang, Hong-Xiu Liu, Yan-Ling Yang, Xing-Juan Xiao, Xiang-Fu Liu, Ning-Guo Liu, Yong-Zhong Lu

**Affiliations:** 1 School of Food and Pharmaceutical Engineering, Guizhou Institute of Technology, Guiyang 550025, China Guizhou Key Laboratory of Agricultural Microbiology, Guizhou Academy of Agricultural Sciences Guiyang China; 2 Guizhou Key Laboratory of Agricultural Microbiology, Guizhou Academy of Agricultural Sciences, Guiyang 550009, China Center of Excellence in Fungal Research, Mae Fah Luang University Chiang Rai Thailand; 3 Center of Excellence in Fungal Research, Mae Fah Luang University, Chiang Rai 57100, Thailand College of Biological Science and Food Engineering, Southwest Forestry University Kunming China; 4 School of Science, Mae Fah Luang University, Chiang Rai 57100, Thailand School of Food and Pharmaceutical Engineering, Guizhou Institute of Technology Guiyang China; 5 College of Biological Science and Food Engineering, Southwest Forestry University, Kunming 650224, China School of Science, Mae Fah Luang University Chiang Rai Thailand; 6 The Key Laboratory of Forest Resources Conservation and Utilization in the South-west Mountains of China Ministry of Education, Key Laboratory of National Forestry and Grassland Administration on Biodiversity Conservation in Southwest China, Yunnan Provincial Key Laboratory for Conservation and Utilization of In-forest Re-source, Key Laboratory of Forest Disaster Warning and Control in Universities of Yunnan Province, Modern Industry School of Edible-fungi, Southwest Forestry University, Kunming 650224, China Key Laboratory of Forest Disaster Warning and Control in Universities of Yunnan Province, Modern Industry School of Edible-fungi, Southwest Forestry University Kunming China

**Keywords:** 2 new taxa, microfungi, new records, Pleosporales, taxonomy

## Abstract

*Miscanthus* is a widely distributed perennial grass in the family Poaceae that hosts a diverse fungal community. During a mycological survey conducted in the Maolan National Nature Reserve, Guizhou Province, China, three fungal isolates were obtained from dead leaves of *Miscanthus sinensis*. Multi-gene phylogenetic analyses of the internal transcribed spacer (ITS), large subunit (LSU), small subunit (SSU), and *tef*1-α sequence data indicated that the new isolates represent two new species, *Periconia guizhouensis* and *Pe. miscanthusensis*, and one new host record of *Paramonodictys globosa*. *Periconia guizhouensis* is distinct from its phylogenetically closest species in having globose to fusiform, guttulate, aseptate conidia with an inconspicuously verrucose surface. *Periconia miscanthusensis* is distinct from its phylogenetically closest species in having apically branched conidiophores produced in acropetal chains and globose, verrucose, aseptate conidia. Comprehensive descriptions, micrographs, and phylogenetic analysis results are provided. Furthermore, these findings enhance understanding of fungi associated with *Miscanthus* and expand current knowledge of fungal diversity in China.

## Introduction

*Miscanthus* belongs to the subtribe Saccharinae, tribe Andropogoneae, subfamily Panicoideae, and family Poaceae (Ge et al. 2017). It is a self-incompatible C4 perennial grass with high photosynthetic efficiency, high biomass yield, and strong environmental adaptability, and it is widely utilized as a biomass energy source and industrial feedstock (Robertson et al. 2017; Wang et al. 2021; Fu et al. 2023). The genus is mainly distributed in eastern Asia, Southeast Asia, and the Pacific islands, with a few species extending into tropical Africa (Sacks et al. 2012). In addition, *Miscanthus* hosts a diverse fungal community. During a fungal survey conducted in the Maolan National Nature Reserve, Guizhou Province, China, several saprophytic fungi resembling *Periconia* and *Paramonodictys* were isolated from dead leaves of *Miscanthus sinensis*.

*Periconia* (Periconiaceae, Pleosporales, Dothideomycetes, Ascomycota; Hyde et al. 2024) was introduced by Tode (1971) with *Pe. lichenoides* as the type species. The sexual morph is characterized by globose ascomata with a central ostiole, fissitunicate asci with short stalks, and broadly fusiform, 1-septate, hyaline ascospores with an entire sheath (Tanaka et al. 2015; Yang et al. 2022a). The asexual morph features macronematous, mononematous conidiophores; monoblastic to polyblastic, terminal or intercalary conidiogenous cells; and ellipsoidal to oblong, catenate or solitary, smooth or verruculose conidia (Ellis 1971; Markovskaja and Kačergius 2014; Hyde et al. 2018; Calvillo-Medina et al. 2020; Su et al. 2023; Pem et al. 2024). Members of *Periconia* are globally distributed and occur as endophytes, saprobes, and pathogens on various hosts, with most associated with graminaceous plants, but they also occur on submerged wood in freshwater habitats (Leukel 1948; Ellis 1971; Romero et al. 2001; Cai et al. 2003; Carmarán and Novas 2003; Markovskaja and Kačergius 2014; Yang et al. 2022a; Su et al. 2023; Shen et al. 2025; Tian et al. 2025; Zhang et al. 2025a).

*Paramonodictys* (Parabambusicolaceae, Pleosporales, Dothideomycetes, Ascomycota) was introduced by Hyde et al. (2020) and typified with *P. solitarius*. The asexual morph is hyphomycetous, characterized by superficial stromata that are subcylindrical or truncated-cone-shaped, lacking conidiophores, bearing monoblastic conidiogenous cells, and producing muriform, globose to subglobose, darkly pigmented conidia (Hyde et al. 2020; Xu et al. 2023; Zhang et al. 2023). The sexual morph remains unknown. All currently known species of *Paramonodictys* are saprophytic, occurring in freshwater and terrestrial habitats on hosts such as *Mangifera*, *Paeonia*, and various types of dead wood (Hyde et al. 2020; Yang et al. 2022b; Li et al. 2023; Xu et al. 2023; Zhang et al. 2023).

During our survey of fungal diversity associated with *Miscanthus* in Maolan, Guizhou Province, we collected three hyphomycetous taxa from the surface of dead leaves of *Miscanthus sinensis*. Based on morphological characteristics and phylogenetic analyses of combined ITS, LSU, SSU, and *tef*1-α sequence datasets, we identified two new species, *Periconia guizhouensis* and *Pe. miscanthusensis*, and recorded a new host association for *Paramonodictys globosa*. Full descriptions, photoplates illustrating macro- and micromorphological characteristics, and phylogenetic trees showing the placement of the new and known taxa are provided in this study.

## Materials and methods

### Sampling and examination of specimens

Specimens were collected from the Maolan National Nature Reserve, Libo County, Guizhou Province, China, at elevations ranging from 490–580 m (25°16'20"–25°16'25" N, 108°2'20"–108°3'60" E). Samples were placed in sterile, moistened plastic bags and transported to the laboratory for detailed examination. Fresh materials were dissected and observed for morphological characteristics, including the structures of conidiophores, conidiogenous cells, and conidia, using stereo microscopes (SMZ 745 and SMZ 800N, Nikon, Tokyo, Japan) and an ECLIPSE Ni compound microscope (Nikon, Tokyo, Japan). Measurements were obtained using Tarosoft Image Framework software, and photoplates were assembled using Adobe Photoshop 2025 (version 26.5; Adobe Systems, CA, USA).

Single-spore isolation was conducted following the protocol of Senanayake et al. (2020). The resulting cultures were incubated at 28 °C under continuous light, and colony morphology was observed and recorded systematically. Voucher specimens were deposited in the Herbarium of the Kunming Institute of Botany (Herb. HKAS), Chinese Academy of Sciences, and living cultures were preserved in the Guizhou Culture Collection (GZCC). Registration numbers from Index Fungorum and the Facesoffungi database (Jayasiri et al. 2015) were obtained for each taxon. The new fungal species were introduced following the guidelines of Chethana et al. (2021).

### DNA extraction, PCR amplification, and sequencing

Fresh mycelia were scraped from PDA plates using sterile toothpicks and transferred into 1.5 mL microcentrifuge tubes. Genomic DNA was extracted using the Ezup Column Fungi Genomic DNA Purification Kit according to the manufacturer’s instructions. PCR amplification targeted four loci: the internal transcribed spacer (ITS), large subunit rDNA (LSU), small subunit rDNA (SSU), and translation elongation factor 1-alpha (*tef*1-α), using the primer pairs ITS5/ITS4 (White et al. 1990), LR0R/LR5 (Vilgalys and Hester 1990), NS1/NS4 (White et al. 1990), and TEF1-983F/TEF1-2218R (Carbone and Kohn 1999), respectively.

PCR amplifications were performed in 25 μL reaction mixtures containing 21 µL of 1.1× T3 Super PCR Mix (Tsingke Biotechnology Co., Ltd., Chengdu), 1 µL of each primer, and 2 µL of genomic DNA. Reactions were run on a JS-G9612 PCR thermocycler (Shanghai Peiqing Technology Co., Ltd., Shanghai, China). Cycling conditions for ITS, LSU, SSU, and *tef*1-α followed the protocols described by Ma et al. (2023). PCR products were visualized on 1% agarose gels and subsequently purified and sequenced by Tsingke Biotechnology Co., Ltd. (Chengdu, China). Newly generated sequences were submitted to GenBank (https://ncbi.nlm.nih.gov/WebSub/).

### Phylogenetic analyses

BioEdit v.7.0.5.3 (Hall 1999) was used to examine raw sequence chromatograms for overall quality, including the detection of base-calling errors, ambiguities, and possible contamination. Forward and reverse reads were assembled using SeqMan v.7.0.0 (DNASTAR, Madison, WI, USA; Swindell and Plasterer 1997). Reference sequences for phylogenetic analyses were obtained from GenBank (Table 1) and downloaded using the One-click Fungal Phylogenetic Tool (OFPT) (Zeng et al. 2023). Each gene region was aligned using the online MAFFT v.7 server, and the alignments were subsequently trimmed and refined with trimAl (Capella-Gutiérrez et al. 2009; Katoh and Standley 2013). Multi-gene alignments were combined using SequenceMatrix 1.7.8 (Vaidya et al. 2011). Maximum likelihood (ML) analyses were performed using the IQ-TREE web server (http://iqtree.cibiv.univie.ac.at/), with the best-fit substitution models automatically tested. Ultrafast bootstrap (BS) analysis was implemented with 1,000 replicates. Maximum likelihood bootstrap values (ML-BS) equal to or greater than 75% are marked near each node. Bayesian inference analyses were conducted using MrBayes v.3.2.7a on the CIPRES web portal (http://www.phylo.org/portal2/, accessed on 9 November 2025). The optimal nucleotide substitution models inferred for each locus were TNe+R3 for LSU, GTR+F+R3 for ITS, TNe+R2 for SSU, and GTR+F+I+G4 for *tef*1-α.

**Table 1.** The table below lists the taxa used in this study, with their respective GenBank accession numbers. **Note**: “T” represents the ex-type strain. “-” indicates data unavailability. Newly generated sequences are represented in bold.

Taxon	Strain	GenBank Accessions			
		SSU	ITS	LSU	*tef*1–α
*Massarina cisti*	CBS 266.62^T^	FJ795490	LC014568	AB807539	AB808514
*M. eburnea*	CBS 473.64	GU296170	OM337528	GU301840	GU349040
*Paramonodictys dispersa*	KUNCC 10782	OQ135187	ON261159	OQ146982	OQ943183
*P. dispersa*	KUNCC 10783	OQ135188	ON261160	OQ146983	OQ943184
*P. dispersa*	KUNCC 10788^T^	OQ135189	ON261165	OQ146988	OQ943185
*P. globosa*	BWL59	–	PX380602	PX380580	–
***P. globosa***	**GZCC 25-0768**	**PX654861**	**PX654855**	**PX654858**	**PX733140**
*P. globosa*	GZCC 23-0594^T^	–	OR139016	OR091331	OR494045
*P. globosa*	ZHKUCC 24-1234	–	PX380601	PX380579	–
*P. hongheensis*	KUMCC 21-0343^T^	ON329821	ON350762	ON329822	OL505582
*P. paeoniae*	CGMCC 3.24437^T^	OR253211	OR253139	OR253298	OR251149
*P. paeoniae*	UESTCC 23.0085	OR253212	OR253140	OR253299	OR251150
*P. solitaria*	GZCC 20-0007^T^	MN901118	MN901152	MN897835	MT023012
*P. yunnanensis*	KUMCC 21-0337^T^	OL436230	OL436231	OL436226	OL505585
*P. yunnanensis*	KUMCC 21-0347	OL436234	OL436233	OL436228	OL505586
*Periconia algeriana*	CBS 321.79^T^	–	MH861212	MH872979	–
*Pe. alishanica*	KUMCC 19-0174	–	MW063167	MW063231	MW183792
*Pe. alishanica*	MFLUCC 19-0145^T^	–	MW063165	MW063229	MW183790
*Pe. alishanica*	NCYUCC 19-0186	–	MW063166	MW063230	MW183791
*Pe. ananasi*	KUMCC 21-0470	OL979226	OM102539	OL985955	OM007977
*Pe. ananasi*	MFLUCC 21-0155^T^	OL606142	OL753685	OL606153	OL912946
*Pe. aquatica*	MFLUCC 16-0912^T^	–	KY794701	KY794705	KY814760
*Pe. arecacearum*	SNT19A	PP639222	PP592462	PP621090	PP828795
*Pe. artemisiae*	KUMCC 20-0265^T^	MW448658	MW448657	MW448571	MW460898
*Pe. atropurpurea*	CBS 381.55	–	MH857524	MH869061	–
*Pe. bambusicola*	ZHKUCC 24-1141	–	PQ276762	PQ276767	–
*Pe. banksiae*	CBS 129526^T^	–	JF951147	NG_064279	–
*Pe. byssoides*	MFLUCC 17-2292	MK347858	MK347751	MK347968	MK360069
*Pe. byssoides*	MFLUCC 19-0134	–	MW063164	MW063228	MW183789
*Pe. byssoides*	MFLUCC 20-0172^T^	–	MW063162	MW063226	–
*Pe. byssoides*	NCYUCC 19-0314	–	MW063163	MW063227	–
*Pe. byssoides*	UESTCC 22.0132	OP956054	OP955985	OP956010	OP961451
*Pe. byssoides*	UESTCC 22.0137	OP956036	OP955967	OP955992	OP961433
*Pe. byssoides*	UESTCC 22.0138	OP956038	OP955969	OP955994	OP961435
*Pe. caespitosa*	LAMIC 110/16^T^	–	MH051906	MH051907	–
*Pe. calamagrostidicola*	CPC 46238^T^	–	PP791432	PP791460	–
*Pe. celtidis*	MFLU 19-2784^T^	–	NR_174830	NG_079543	–
*Pe. chengduensis*	CGMCC 3.23930^T^	OP956056	OP955987	OP956012	OP961453
*Pe. chengduensis*	UESTCC 22.0140	OP956046	OP955977	OP956002	OP961443
*Pe. chengduensis*	UESTCC 22.0142	OP956047	OP955978	OP956003	OP961444
*Pe. chiangraiensis*	KUMCC 21-0471	OL979227	OM102540	OL985956	OM007978
*Pe. chiangraiensis*	MFLUCC 21-0164	OL606143	OL753686	OL606154	OL912947
*Pe. chimonanthi*	KUMCC 20-0266^T^	MW448656	NR_176752	MW448572	MW460897
*Pe. chimonanthi*	UESTCC 22.0133	OP956033	OP955964	OP955989	OP961430
*Pe. chimonanthi*	UESTCC 22.0144	OP956043	OP955974	OP955999	OP961440
*Pe. circinata*	CBS 263.37	–	MW810265	MH867413	MW735660
*Pe. citlaltepetlensis*	ENCB 140251^T^	–	MH890645	MT625978	–
*Pe. citlaltepetlensis*	IOM 325319.2	–	MT649221	MT649216	–
*Pe. cookei*	MFLUCC 17-1399	–	MG333490	MG333493	MG438279
*Pe. cookei*	MFLUCC 17-1679	–	–	MG333492	MG438278
*Pe. cortaderiae*	MFLUCC 15-0451	KX986346	KX965734	KX954403	KY429208
*Pe. cortaderiae*	MFLUCC 15-0453	–	KX965733	KX954402	KY320574
*Pe. cortaderiae*	MFLUCC 15-0457^T^	KX986345	KX965732	KX954401	KY310703
*Pe. cynodontis*	CGMCC 3.23927^T^	OP909920	OP909925	OP909921	OP961434
*Pe. cyperacearum*	CPC 32138^T^	–	NR_160357	NG_064549	–
*Pe. delonicis*	MFLUCC 17-2584^T^	MK347832	–	MK347941	MK360071
*Pe. dicranopteridis*	HKAS 129916	PV138560	PV138731	PV138587	PV177119
*Pe. didymosporum*	MFLU 15-0058^T^	KP761738	KP761734	KP761731	KP761728
*Pe. digitata*	CBS 510.77	AB797271	LC014584	AB807561	AB808537
*Pe. dujuanhuensis*	KUNCC 23-13482^T^	–	PQ340469	PP189907	PQ456957
*Pe. elaeidis*	MFLUCC 17-0087^T^	MH108551	MG742713	MH108552	–
*Pe. endophytica*	ZHKUCC 23-0995^T^	NG_243017	OR995582	OR995588	PP025968
*Pe. endophytica*	ZHKUCC 23-0996	PP277723	OR995583	OR995589	PP025969
*Pe. epilithographicola*	CBS 144017^T^	–	NR_157477	–	–
*Pe. epilithographicola*	MFLUCC 21-0153	OL606144	OL753687	OL606155	OL912948
*Pe. festucae*	CGMCC 3.23929^T^	OP956042	OP955973	OP955998	OP961439
*Pe. floridana*	CPC 45904^T^	–	PP791420	PP791448	–
*Pe. genistae*	CBS 322.79^T^	–	MH861213	MH872980	–
*Pe. guangxiense*	GZAAS 25-0735^T^	PX118890	PX118886	PX118882	PX522310
*Pe. guangxiense*	GZAAS 25-0733	PX118891	PX118887	PX118883	PX522311
***Pe. guizhouensis***	**GZCC 25-0766^T^**	**PX654862**	**PX654856**	**PX654859**	**PX733136**
***Pe. guizhouensis***	**GZCC 25-27613**	**PX716655**	**PX723935**	**PX723941**	**PX733137**
*Pe. homothallica*	CBS 139698^T^	AB797275	AB809645	NG_059397	AB808541
*Pe. hongheensis*	KUNCC 23-13549	–	PQ340466	PP189904	–
*Pe. hongheensis*	KUNCC 23-13550^T^	–	PQ340467	PP189905	PQ456958
*Pe. hydei*	ZHKUCC 24-2101	PV437586	PV434171	PV436640	PV588047
*Pe. hydei*	ZHKUCC 24-2102	PV437587	PV434172	PV436641	PV588048
*Pe. hydeiguttulosa*	UESTCC 23.0456	PQ184747	PQ394046	PQ184695	PQ346487
*Pe. hydeiguttulosa*	UESTCC 24.0232	PQ184755	PQ394070	PQ184717	PQ346515
*Pe. igniaria*	CBS 298.66	–	MH858798	MH870438	–
*Pe. igniaria*	CBS 583.66	–	MH858888	MH870553	–
*Pe. imperatae*	CGMCC 3.23931^T^	OP956053	OP955984	OP956009	OP961450
*Pe. imperatae*	UESTCC 22.0145	OP956048	OP955979	OP956004	OP961445
*Pe. imperatae*	UESTCC 22.0146	OP956052	OP955983	OP956008	OP961449
*Pe. kunmingensis*	MFLUCC 18-0679	OR225814	MH892346	MH892399	MH908963
*Pe. lateralis*	CBS 292.36	–	MH855804	MH867311	–
*Pe. linzhiensis*	HKAS 144526^T^	PQ675368	PQ684989	PQ675408	PQ671465
*Pe. linzhiensis*	HKAS 144527	PQ675369	PQ684990	PQ675409	PQ671466
*Pe. macrospinosa*	CBS 135663	KP184080	KP183999	KP184038	–
*Pe. minutissima*	MFLUCC 15-0245	–	KY794703	KY794707	–
***Pe. miscanthusensis***	**GZCC 25-0767^T^**	**PX654863**	**PX654857**	**PX654860**	**PX733138**
***Pe. miscanthusensis***	**GZCC 25-27614**	**PX716656**	**PX723936**	**PX723942**	**PX733139**
*Pe. motuoensis*	KUNCC 24-17924	PQ438116	PQ373177	PQ438054	PQ661220
*Pe. motuoensis*	KUNCC 24-17925	PQ438117	PQ373178	PQ438055	PQ661221
*Pe. motuoensis*	KUNCC 24-17926	PQ438114	PQ373175	PQ438052	PQ661218
*Pe. motuoensis*	KUNCC 24-17927	PQ438115	PQ373176	PQ438053	PQ661219
*Pe. muchuanensis*	CGMCC 3.25599^T^	NG_244216	NR_199245	NG_244340	PQ278553
*Pe. neobrittanica*	CPC 37903^T^	–	MN562149	MN567656	–
*Pe. neohongheensis*	ZHKUCC 24-1140	PQ269745	PQ269733	PQ269739	PQ362733
*Pe. neominutissima*	CBS 149514^T^	–	OQ628478	OQ629060	–
*Pe. palmicola*	MFLUCC14-0400^T^	MN648319	–	MN648327	MN821070
*Pe. penniseti*	CGMCC 3.23928^T^	OP956040	OP955971	OP955996	OP961437
*Pe. philadelphiana*	CBS H-25197^T^	–	OQ628486	OQ629068	–
*Pe. prolifica*	CBS 209.64^T^	–	NR_160097	MH870050	–
*Pe. pseudobyssoides*	DLUCC 0850	–	MG333491	MG333494	MG438280
*Pe. pseudobyssoides*	H4151	AB797278	LC014587	AB807568	AB808544
*Pe. pseudobyssoides*	MAFF 243874	AB797270	LC014588	AB807560	AB808536
*Pe. pseudobyssoides*	UESTCC 22.0135	OP956034	OP955965	OP955990	OP961431
*Pe. pseudobyssoides*	UESTCC 22.0147	OP956044	OP955975	OP956000	OP961441
*Pe. pseudodigitata*	CBS 139699^T^	AB797274	LC014591	AB807564	AB808540
*Pe. pseudodigitata*	JCM 13164	AB797272	LC014589	AB807562	AB808538
*Pe. pseudodigitata*	JCM 13165	AB797273	LC014590	AB807563	AB808539
*Pe. sahariana*	CBS 320.79^T^	–	MW444854	MH872978	–
*Pe. salina*	GJ374^T^	MN017912	MN047086	MN017846	–
*Pe. shannanensis*	HKAS 134945^T^	PP968558	PP968552	PP968555	PQ226770
*Pe. sichuanensis*	CGMCC 3.25598^T^	PQ066545	PQ067867	NG_244339	PQ278551
*Pe. spodiopogonis*	CGMCC 3.23932^T^	OP956032	OP955963	OP955988	OP961429
*Pe. submersa*	MFLUCC 16-1098^T^	–	KY794702	KY794706	KY814761
*Pe. thailandica*	MFLUCC 17-0065^T^	KY753889	KY753887	KY753888	–
*Pe. thysanolaenae*	KUMCC 20-0262^T^	NG_081407	MW442967	MW444850	MW460896
*Pe. variicolor*	CBS 120374^T^	–	DQ336713	–	–
*Pe. verrucosa*	MFLUCC 17-2158^T^	MT226686	MT310617	MT214572	MT394631
*Pe. verrucosa*	UESTCC 22.0136	OP956035	OP955966	OP955991	OP961432
*Pe. verrucosa*	UESTCC 22.0148	OP956039	OP955970	OP955995	OP961436
*Pe. wurfbainiae*	ZHKUCC 23-0999^T^	NG_243019	OR995586	OR995592	PP025972
*Pe. wurfbainiae*	ZHKUCC 23-1000	PP277727	OR995587	OR995593	PP025973
*Pe. xishuangbannaensis*	GZAAS25-0707^T^	PX118892	PX118888	PX118884	PX522312
*Pe. xishuangbannaensis*	GZAAS25-0734	–	PX118889	PX118885	–
*Pe. yangjiangensis*	ZHKUCC 23-0997^T^	NG_243018	OR995584	OR995590	PP025970
*Pe. yangjiangensis*	ZHKUCC 23-0998	PP277725	OR995585	OR995591	PP025971
*Pe. yantingensis*	SICAUCC 23-0047^T^	PP003824	PP060663	PP057956	PP061142
*Pe. yantingensis*	SICAUCC 23-0147	PP853378	PP844875	PP826165	PP850053
*Pe. yunnanensis*	KUNCC 23-14259^T^	–	PQ340468	PP189906	PQ456960
*Pe. yunnanensis*	KUNCC 23-16910	–	PQ607755	PP189925	PQ456959

The phylogenetic tree was visualized and edited using FigTree v.1.4.0, and the final graphic design and figure layouts were completed using Adobe Photoshop 2024 and Adobe Illustrator 2021 (Adobe Systems, San Jose, CA, USA).

## Results

### Phylogenetic analysis

Partial ITS, LSU, SSU, and *tef*1-α nucleotide sequences were used to determine the phylogenetic positions of the new collections. The dataset comprised sequences from 134 isolates representing 71 *Periconia* and six *Paramonodictys* species. *Massarina cisti* (CBS 266.62) and *M. eburnea* (CBS 473.64) were selected as the outgroup taxa. The concatenated sequence matrix included ITS (1–511 bp), LSU (512–1,349 bp), SSU (1,350–2,347 bp), and *tef*1-α (2,348–3,240 bp), with gaps included. The concatenated ITS, LSU, SSU, and *tef*1-α datasets were analyzed using both maximum likelihood (ML) and Bayesian inference (BI) methods, which yielded similar tree topologies.

**Figure 1.**
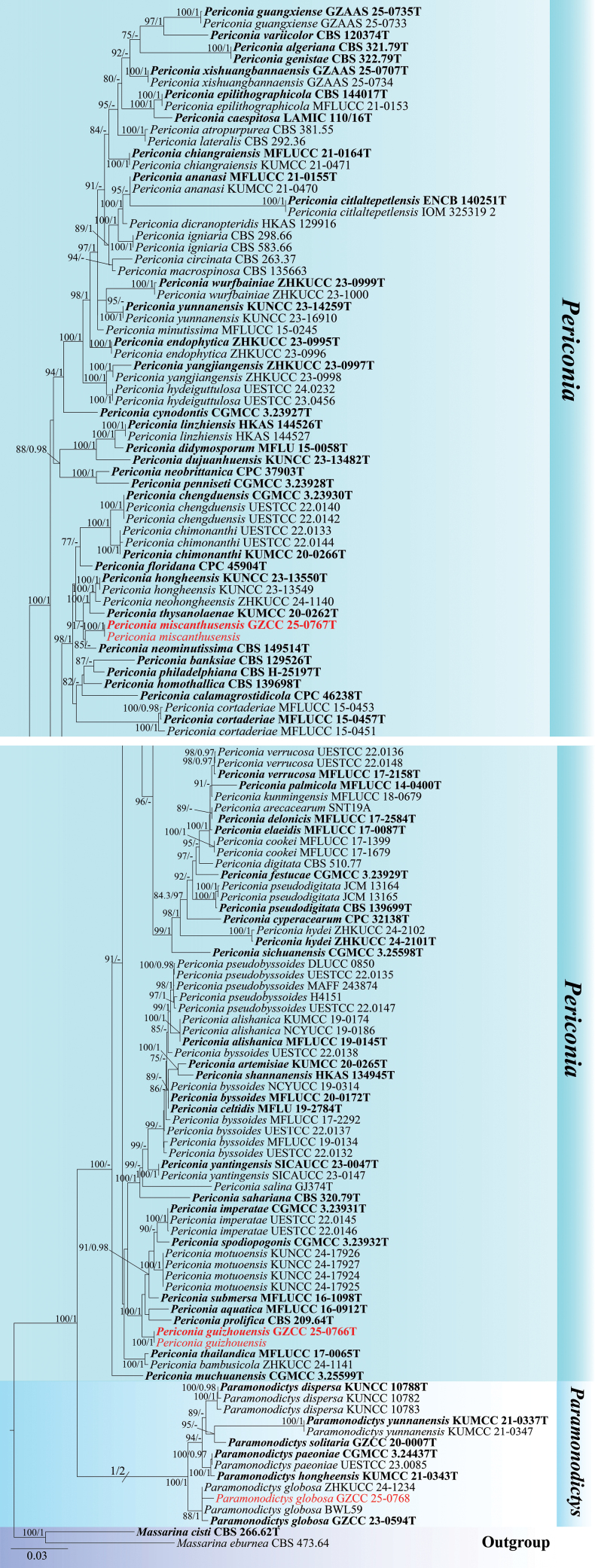
Maximum likelihood (ML) majority-rule consensus tree based on ITS, LSU, SSU, and *tef*1-α sequence data. ML bootstrap support values (ML-BS ≥ 75%) and Bayesian posterior probabilities (BYPP ≥ 0.95) are indicated below or above the nodes. Ex-type strains are shown in bold and marked with T, and newly generated sequences are shown in red.

Phylogenetic relationships among Pleosporales species were well resolved in the multi-gene phylogenetic analysis. The resulting phylogenetic tree confirmed that the two newly obtained isolates of *Periconia guizhouensis* (GZCC 25-0766 and GZCC 25-27613) formed a distinct clade. They clustered with *Pe. aquatica* (MFLUCC 16-0912), *Pe. imperatae* (CGMCC 3.23931, UESTCC 22.0145, UESTCC 22.0146), *Pe. motuoensis* (KUNCC24-17925, KUNCC24-17924, KUNCC24-17927, KUNCC24-17926), *Pe. prolifica* (CBS 209.64), *Pe. spodiopogonis* (CGMCC 3.23932), and *Pe. submersa* (MFLUCC 16-1098), supported by 100% MLB/1.00 BYPP. Likewise, two isolates of *Pe. miscanthusensis* (GZCC 25-0767 and GZCC 25-27614) clustered together and were sister to *Pe. neominutissima* (CBS 149514), supported by 85% ML-BS (Fig. 1). Additionally, *Paramonodictys globosa* (GZCC 25-0768) grouped with *P. globosa* (GZCC 23-0594, BWL59, and ZHKUCC 24-1234), receiving 88% ML-BS/1.00 BYPP support.

### Taxonomy

#### Paramonodictys globosa

Taxon classificationFungiPleosporalesParabambusicolaceae

Jian Ma, Y.Zhe Zhang & Y.Z. Lu

CB390802-B774-554D-AC26-273AEF29B362

Fungal Names: FN 571668

Facesoffungi Number: FoF19046

Fig. 2

Paramonodictys globosa Jian Ma, Y.Zhe Zhang & Y.Z. Lu, in Zhang, Chen, Ma, Lu, Chen and Liu, Frontiers Microbiol. 14 (no. 1253239): 9 (2023).

##### Description.

***Saprobic*** on dead leaves of *Miscanthus sinensis*. Sexual morph: Undetermined. Asexual morph: Hyphomycetous. ***Colonies*** on natural substrate effuses, superficial, black. ***Mycelium*** partly immersed, branched, septate, smooth, hyaline to pale brown. ***Conidiophores*** are absent. ***Conidiogenous cells*** holoblastic, integrated, terminal, cylindrical, short, hyaline to brown, often arising directly on the superficial mycelium. ***Conidia*** 35–65 × 35–50 µm (x̄ = 50 × 44 μm, n = 30), solitary, irregular subglobose or globose, hyaline to olivaceous brown to dark brown, muriform, thickened and darkened at the septa.

**Figure 2.**
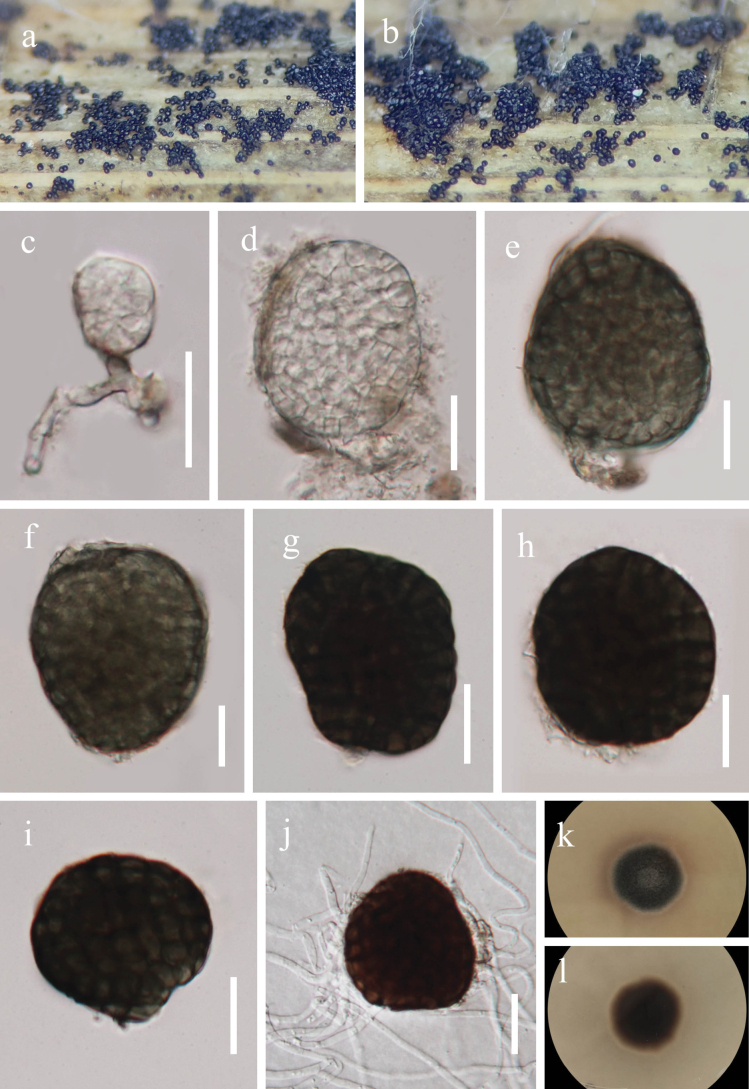
*Paramonodictys globosa* (GZAAS 25-07813) **a, b**. Colonies on host surface; **c**. Conidiophores, conidiogenous cells, and conidia; **d–i**. Conidia; **j**. Germinated conidium; **k, l**. Colony on PDA (above and below). Scale bars: 20 μm (**c–j**).

##### Cultural characteristics.

Conidia germinating on PDA medium within 24 h and germ tube produced from around of conidium. Colonies on PDA medium reaching 15 mm diam in 10 days at 28 °C in natural light, dense, circular, with entire edge, olivaceous brown; reverse brown.

##### Material examined.

China, • Guizhou Province, Qiannan Buyi and Miao Autonomous Prefecture, Libo County, Guizhou Maolan National Nature Reserve, 25°16'23"N, 108°3'56"E, on dead leaves of *Miscanthus sinensis*, 31 May 2025, Xingjuan Xiao, MLM5 (GZAAS 25-07813), living culture GZCC 25-0768.

##### Notes.

*Paramonodictys globosa* was introduced by Zhang et al. (2023) from submerged decaying wood in a freshwater stream in Guangxi Zhuang Autonomous Region, China. Our collection was obtained from dead leaves of *Miscanthus sinensis* in a terrestrial habitat in Guizhou, China. Morphologically, our specimen is similar to *P. globosa* (HKAS 129169) in having solitary, irregularly subglobose or globose, muriform conidia with thickened and darkened septa (35–65 × 35–50 µm vs. 34–65 × 24–60 µm) (Zhang et al. 2023). Moreover, comparisons of ITS, LSU, and *tef*1-α sequences revealed differences of only 4 bp (1 gap, total 606 bp), 4 bp (0 gaps, total 822 bp), and 16 bp (no gaps, total 887 bp) between our strain GZCC 25-0768 and the ex-type strain (GZCC 23-0594) of *P. globosa*, respectively. Based on the combined morphological characteristics and molecular evidence, we identify our collection as *Paramonodictys globosa*, representing the first report of this species from *M. sinensis*.

#### Periconia guizhouensis

Taxon classificationFungiPleosporalesPericoniaceae

L.J. Zhang, Y.Z. Lu & X.J. Xiao sp. nov.

0908E488-FA7B-5766-9D6B-F6B2CBDB4A47

Index Fungorum: IF904704

Facesoffungi Number: FoF19047

Fig. 3

##### Etymology.

Referring to the collecting location at Guizhou Province, where the holotype was discovered.

##### Holotype.

GZAAS 25-07814

##### Description.

***Saprobic*** on dead leaves of *Miscanthus sinensis*. Sexual morph: Undetermined. Asexual morph: ***Colonies*** on natural substrate effuse, superficial, hairy, dark brown to black. ***Mycelium*** immersed, branched, septate, smooth, brown to dark brown. ***Conidiophores*** 400–750 × 12–22 μm (x̄ = 630 × 18 µm, n = 20), macronematous, mononematous, erect, straight to slightly curved, solitary, cylindrical, apically branched, brown to dark brown, multi-septate, smooth-walled. ***Conidiogenous cells*** 6–16 × 5–10 μm (x̄ = 10 × 7 µm, n = 20), polyblastic, terminal at the apex, ovoid to subglobose, light to dark brown. ***Conidia*** 12–18 × 7–10 μm (x̄ = 15 × 8.5 µm, n = 30), solitary or in short chains, globose to fusiform, guttulate, smooth to verrucose, aseptate, pale brown to dark brown.

**Figure 3.**
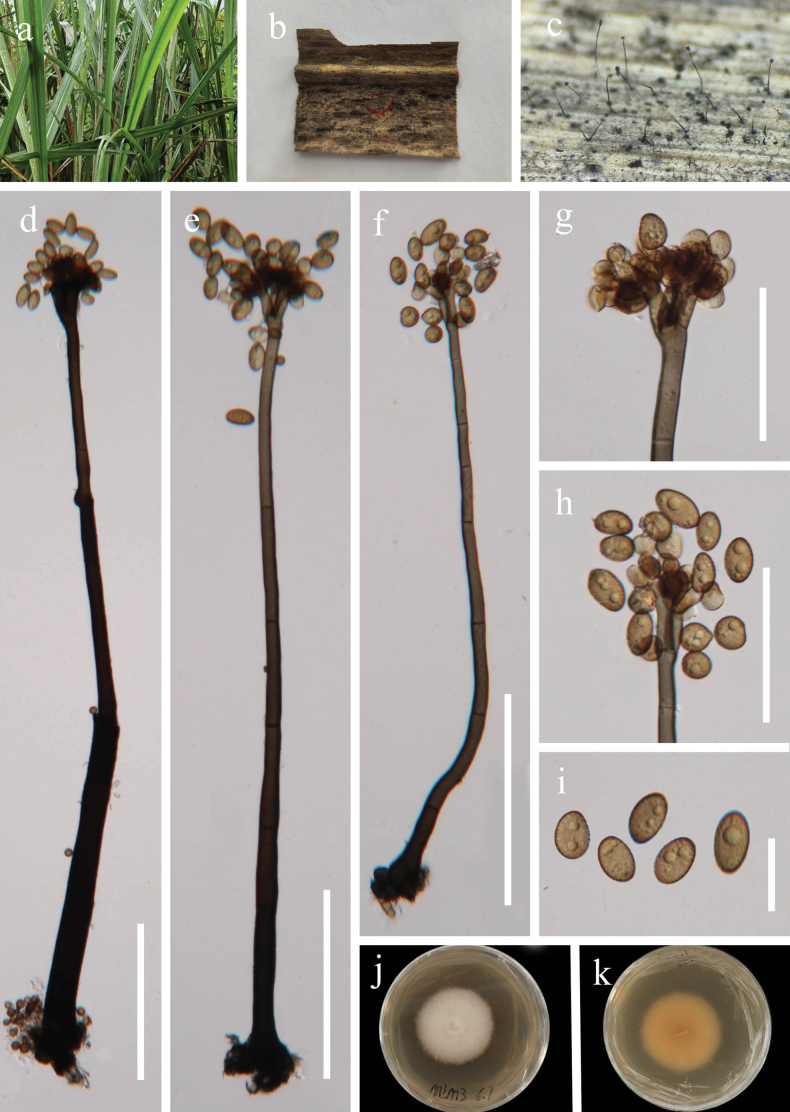
*Periconia guizhouensis* (GZAAS 25-07814, holotype). **a**. Host of *Miscanthus sinensis*; **b**. Colonies on a decaying leaf of *Miscanthus sinensis*; **c**. Conidiophores on host surface; **d–f**. Conidiophore, conidiogenous cells, and conidia; **g, h**. Conidiogenous cells and conidia; **i**. Conidia; **j, k**. Colony on PDA (above and below). Scale bars: 100 μm (**d–f**); 50 μm (**g, h**); 20 μm (**i**).

##### Cultural characteristics.

Conidia germinated on PDA medium within 12 h, and germ tubes produced from around of conidium. Colonies on PDA medium reaching 33 mm diam in 10 days at 28 °C in natural light, dense, circular, with entire edge, white reverse pale brown.

##### Material examined.

China, • Guizhou Province, Qiannan Buyi and Miao Autonomous Prefecture, Libo County, Guizhou Maolan National Nature Reserve, 25°16'23"N, 108°3'56"E, on dead leaves of *Miscanthus sinensis*, 31 May 2025, Xingjuan Xiao, MLM3 (GZAAS 25-07814, holotype), ex-type living culture GZCC 25-0766, other living culture GZCC 25-27613.

##### Notes.

In the phylogenetic tree, *Periconia guizhouensis* (GZCC 25-0766 and GZCC 25-27613) formed a distinct lineage within *Periconia* and clustered with *Pe. prolifica* (CBS 209.64) and *Pe. aquatica* (MFLUCC 16-0912) (Fig. 1). The ITS and LSU sequences of *Pe. guizhouensis* (GZCC 25-0766, type) differ from those of *Pe. prolifica* (CBS 209.64, type) by 23 bp (498/535 bp, 14 gaps) and 3 bp (854/857 bp, 0 gaps), respectively; *Pe. prolifica* lacks SSU and *tef*1-α sequences. *Periconia guizhouensis* (GZCC 25-0766, type) and *Pe. aquatica* (MFLUCC 16-0912, type) differ by 12 bp (438/452 bp, 2 gaps) in ITS, 3 bp (786/789 bp, 0 gaps) in LSU, and 25 bp (809/834 bp, 0 gaps) in *tef*1-α; *Pe. aquatica* lacks an SSU sequence. Morphologically, *Pe. prolifica* shares similar characteristics with our collection in having subglobose, aseptate conidia. However, *Pe. guizhouensis* can be readily distinguished by its verrucose conidia, whereas the conidia of *Pe. prolifica* are consistently smooth-walled (Bunning and Griffiths 1982; Markovskaja and Kačergius 2014). *Periconia guizhouensis* differs from *Pe. aquatica* in that the latter has unbranched, smaller conidiophores (400–750 × 12–22 μm vs. *Conidiophores* 370–454 × 8.5–11 μm) and smaller conidia (12–18 × 7–10 μm vs. 10–12 × 6–7 µm) with more obviously minutely verruculose to shortly echinulate ornamentation (Hyde et al. 2017). Moreover, *Periconia guizhouensis* can be easily distinguished from *Pe. imperatae*, *Pe. motuoensis*, *Pe. spodiopogonis*, and *Pe. submersa* by its globose to fusiform, guttulate, aseptate conidia with inconspicuous verrucosity (Hyde et al. 2017; Su et al. 2023; Sun et al. 2025). Therefore, based on morphological characteristics and molecular evidence, *Periconia guizhouensis* is introduced as a new species.

#### Periconia miscanthusensis

Taxon classificationFungiPleosporalesPericoniaceae

L.J. Zhang, Y.Z. Lu & X.J. Xiao sp. nov.

43E00B18-B573-52AA-80C7-DADEAB68B746

Index Fungorum: IF904705

Facesoffungi Number: FoF19048

Fig. 4

##### Etymology.

The specific epithet miscanthusensis refers to *Miscanthus*, the grass genus from which the type strain was isolated.

##### Holotype.

GZAAS 25-07815

##### Description.

***Saprobic*** on dead leaves of *Miscanthus sinensis*. Sexual morph: Undetermined. Asexual morph: ***Colonies*** on natural substrate effuse, superficial, hairy, dark brown to black. ***Mycelium*** immersed, branched, septate, smooth, brown. ***Conidiophores*** 350–900 × 10–20 μm (x̄ = 540 × 14 µm, n = 20), macronematous, mononematous, erect, straight to slightly curved, cylindrical, unbranched, brown to dark brown, multi-septate, smooth-walled. Typically, a single longer conidiophore is present on the stroma, accompanied by 1–2 shorter conidiophores 70–125 × 4–17 μm (x̄ = 98 × 7 µm, n = 20). ***Conidiogenous cells*** 7–14 × 6–12 μm (x̄ = 11 × 9 µm, n = 20), polyblastic, proliferous, terminal at the apex, globose to subglobose, light to dark brown. ***Conidia*** 4–9 × 4–7 μm (x̄ = 6 × 5.5 µm, n = 30), compact chains, globose, smooth to verrucose, aseptate, pale brown to dark brown.

**Figure 4.**
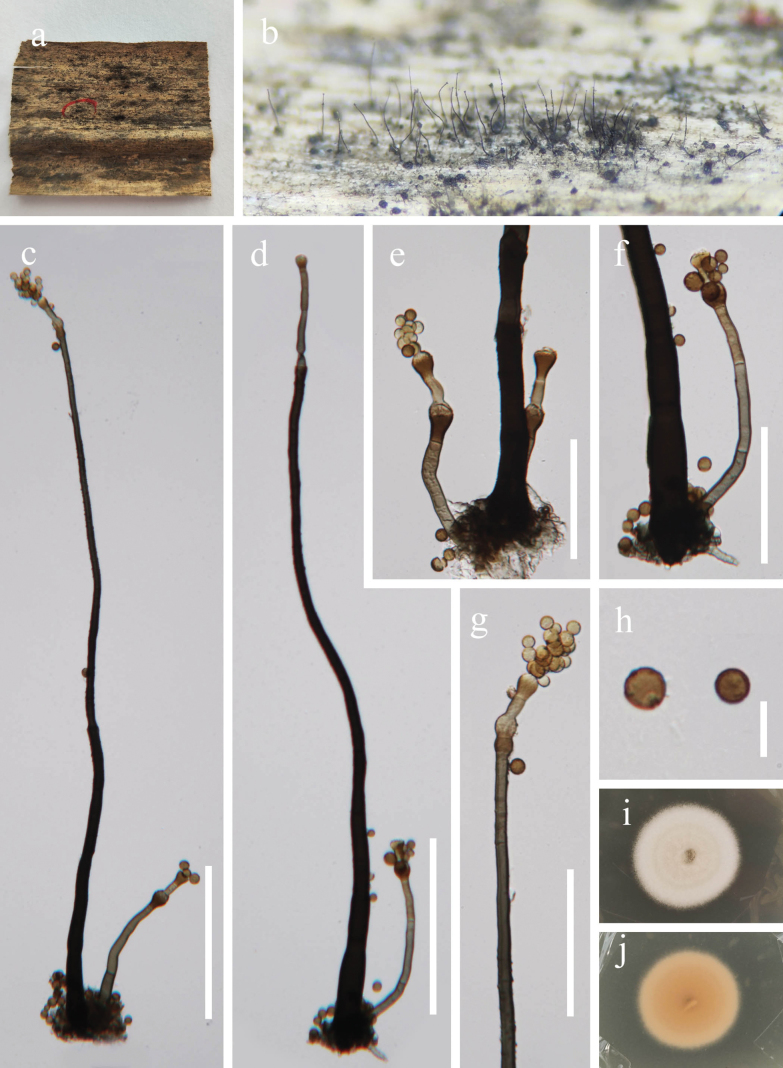
*Periconia miscanthusensis* (GZAAS 25-07815, holotype). **a**. Colonies on a decaying leaf of *Miscanthus sinensis*; **b**. Conidiophores on host surface; **c–f**. Conidiophore, conidiogenous cells, and conidia; **g**. Conidiogenous cells and conidia; **h**. Conidia; **i, j**. Colony on PDA (above and below). Scale bars: 100 μm (**c, d**); 50 μm (**e–g**); 10 μm (**h**).

##### Cultural characteristics.

Conidia germinated on PDA medium within 12 h, and germ tubes produced from around of conidium. Colonies on PDA medium reaching 18 mm diam in 10 days at 28 °C in natural light, dense, circular, with entire edge, white reverse pale brown.

##### Material examined.

China, • Guizhou Province, Qiannan Buyi and Miao Autonomous Prefecture, Libo County, Guizhou Maolan National Nature Reserve, 25°16'23"N, 108°3'56"E, on dead leaves of *Miscanthus sinensis*, 31 May 2025, Xingjuan Xiao, MLM4 (GZAAS 25-07815, holotype), ex-type living strain GZCC 25-0767, other living culture GZCC 25-27614.

##### Notes.

Phylogenetic analyses revealed that our strains (GZCC 25-0767 and GZCC 25-27614) formed a distinct clade and were sister to *Periconia neominutissima* (CBS 149514) (Fig. 1). Pairwise nucleotide comparisons between *Pe. miscanthusensis* (GZCC 25-0767, type) and *Pe. neominutissima* (CBS 149514, type) showed sequence similarities of 95% (480/503 bp, 9 gaps) in ITS and 99% (835/843 bp, 0 gaps) in LSU, indicating significant genetic differences between the two species. Morphologically, *Pe. miscanthusensis* resembles *Pe. miscanthusensis* in having apically branched conidiophores that produce acropetal chains and globose, verrucose, aseptate conidia. However, our species differs by possessing longer conidiophores (350–900 × 10–20 μm vs. 700 × 12–15 µm), larger conidiogenous cells (7–14 × 6–12 μm vs. 6–8 × 4–5 µm), and a single prominent conidiophore accompanied by 1–2 shorter conidiophores (Crous et al. 2023). Therefore, based on morphological characteristics and molecular evidence, *Periconia miscanthusensis* is introduced as a novel species.

## Discussion

Nannizzi (1934) established Periconiaceae with *Periconia* as the type genus, but the family was overlooked in modern taxonomic treatments, and its members were traditionally placed in Massarinaceae. Tanaka et al. (2015) revived this family based on phylogenetic analyses and accepted four genera, *Bambusistroma*, *Flavomyces*, *Noosia*, and *Periconia*. Later, Yang et al. (2022b) re-evaluated *Bambusistroma* and *Noosia* based on morphological comparisons and multi-gene phylogenetic analyses and subsequently synonymized both genera under *Periconia*. Consequently, Periconiaceae currently comprises only two genera, *Flavomyces* and *Periconia* (Hyde et al. 2024). However, because *Flavomyces* lacks clear morphological diagnostic traits and clusters with *Periconia* species in phylogenetic analyses, its generic status remains uncertain and requires further clarification. According to Species Fungorum (https://www.speciesfungorum.org/Names/Names.asp, accessed on 20 November 2025), *Periconia* contains 206 epithets. Among these, 29 species have been transferred to other genera within Dothideomycetes, Pezizomycetes, Sordariomycetes, and Ascomycota genera incertae sedis, and seven species have been synonymized under other *Periconia* species (Persoon 1801; Mason and Ellis 1953; Benjamin and Hesseltine 1959; Ellis 1971; Illman and White 1984; Okada et al. 2000; Partridge and Morgan-Jones 2002; Schubert et al. 2007; Linnakoski et al. 2012; Yang et al. 2022b; Zhang et al. 2025b).

During the past three years, 30 species of *Periconia* have been reported (Su et al. 2023; Cai et al. 2024; Liao et al. 2024; Phookamsak et al. 2024; He et al. 2025; Sun et al. 2025; Wijesinghe et al. 2025; Zhang et al. 2025a; Zhang et al. 2025b). Most species in the genus have been accepted solely based on morphological characters. Molecular data are available for only 68 species, and for the majority of these species, only ITS and LSU sequences have been generated, whereas more informative markers (e.g., mtSSU, *rpb*1, *rpb*2, *tef*1-α, and tub2, remain unavailable (Yang et al. 2022b; Liao et al. 2024; Sun et al. 2025; Wijesinghe et al. 2025; Zhang et al. 2025b). Moreover, several *Periconia* species exhibit noticeable intraspecific variation, including *P. byssoides*, *P. cortaderiae*, *P. prolifica*, and *P. pseudobyssoides* (Yang et al. 2022b). Therefore, future studies require additional phylogenetic markers to more accurately resolve species boundaries within this highly diverse genus (Yang et al. 2022b; Liao et al. 2024; He et al. 2025; Zhang et al. 2025a).

Most *Periconia* species have been recorded from temperate and tropical regions (Numata et al. 1997; Kolomiets et al. 2008; Sarkar et al. 2019; Gunasekaran et al. 2021; Liu et al. 2024; Madagammana et al. 2024; He et al. 2025). The genus is widely distributed and has been reported as endophytes, plant pathogens, and saprobes across terrestrial, mangrove, marine, and freshwater habitats (Odvody et al. 1977; Romero et al. 2001; Alias and Jones 2000; Goga 2000; Kohlmeyer 1977; Luo et al. 2004; Liu et al. 2017a, b; Hyde et al. 2017; Jayasiri et al. 2019; Sarkar et al. 2019; Shen et al. 2025; Bao et al. 2025; Hongsanan et al. 2025; Tian et al. 2025). In addition to their ecological diversity, species of this genus produce secondary metabolites with diverse biological activities, including antimicrobial, anti-human immunodeficiency virus (HIV), cytotoxic, and anti-inflammatory effects (Numata et al. 1997; Teles et al. 2006; Bhilabutra et al. 2007; Wu et al. 2015a, b; Liu et al. 2017b; Azhari and Supratman 2021). These findings underscore the ecological and biochemical significance of *Periconia* and highlight the need for continued taxonomic and ecological investigations of newly discovered species.

*Periconia prolifica* has been reported from a wide variety of substrates in Saudi Arabia, including driftwood in Jeddah, mangrove wood, and material from the Red Sea coast, as well as seawater and sea foam along the Arabian Gulf coast (Aleem 1979; Bunning and Griffiths 1982; Bokhary et al. 1992; Vishwakiran et al. 2001; Hodhod 2013; Hodhod et al. 2023). It has also been recorded from diverse geographical regions worldwide, including Brazil, China, Egypt, Ghana, Indonesia, Japan, Kuwait, Malaysia, Mexico, Saudi Arabia, South Africa, and the USA, on substrates such as decayed intertidal mangrove wood and seedlings (Aleem 1979; Vrijmoed et al. 1982; Vishwakiran et al. 2001; Abdel-Wahab 2005; Pang et al. 2011; Hodhod et al. 2023). However, its taxonomic position has long been controversial. The sexual morph of *Pe. prolifica* was originally classified in *Halosphaeria* as *H. cucullata* (Kohlm.) Kohlm. (Kohlmeyer 1972) but was later transferred to *Okeanomyces* (Halosphaeriaceae, Microascales) by Pang et al. (2004) based on its conidiogenetic features. In this study, the ex-type strain of *Pe. prolifica* formed a sister clade with our newly collected species, *Pe. guizhouensis* (GZCC 25-0766 and GZCC 25-27613) (Fig. 1). Morphologically, our species produces acropetal conidial chains from conidiogenous cells on conidiophores, whereas *Pe. prolifica* forms basipetal conidial chains directly on the wood substrate (Bunning and Griffiths 1982; Carmarán and Novas 2003; Hodhod et al. 2023). These morphological differences may reflect adaptations to distinct habitats. Thus, both multi-gene phylogenetic analyses and detailed morphological observations are essential for robust fungal taxonomy.

## Supplementary Material

XML Treatment for Paramonodictys globosa

XML Treatment for Periconia guizhouensis

XML Treatment for Periconia miscanthusensis

## Additional information

### Use of AI

No use of AI was reported.

### Author ORCIDs

Li-Juan Zhang https://orcid.org/0000-0002-3234-6757

Hong-Xiu Liu https://orcid.org/0009-0004-9311-9779

Yan-Ling Yang https://orcid.org/0009-0006-9574-8194

Xing-Juan Xiao https://orcid.org/0009-0003-8769-4534

Xiang-Fu Liu https://orcid.org/0000-0003-0100-2094

Ning-Guo Liu https://orcid.org/0000-0002-9169-2350

Yong-Zhong Lu https://orcid.org/0000-0002-1033-5782

### Data availability

All of the data that support the findings of this study are available in the main text.
